# Prognostic potentials of AI in ophthalmology: systemic disease forecasting via retinal imaging

**DOI:** 10.1186/s40662-024-00384-3

**Published:** 2024-05-06

**Authors:** Yong Yu Tan, Hyun Goo Kang, Chan Joo Lee, Sung Soo Kim, Sungha Park, Sahil Thakur, Zhi Da Soh, Yunnie Cho, Qingsheng Peng, Kwanghyun Lee, Yih-Chung Tham, Tyler Hyungtaek Rim, Ching-yu Cheng

**Affiliations:** 1 Cork University Hospital, Cork, Ireland; 2 Division of Retina, Severance Eye Hospital, Yonsei University College of Medicine, Seoul, South Korea; 3 Division of Cardiology, Severance Cardiovascular Hospital, Yonsei University College of Medicine, Seoul, South Korea; 4 Singapore Eye Research Institute, Singapore National Eye Centre, Singapore, Singapore; 5 Department of Ophthalmology, Yong Loo Lin School of Medicine, National University of Singapore, Singapore, Singapore; 6 Mediwhale Inc, Seoul, Republic of Korea; 7 Department of Education and Human Resource Development, Seoul National University Hospital, Seoul, South Korea; 8 Department of Ophthalmology, National Health Insurance Service Ilsan Hospital, Goyang, Republic of Korea; 9 Centre for Innovation and Precision Eye Health, Yong Loo Lin School of Medicine, National University of Singapore, Singapore, Singapore; 10 Ophthalmology and Visual Sciences Academic Clinical Program, Singapore, Singapore

**Keywords:** Artificial intelligence, Deep learning, Systemic disease, Cardiovascular disease, Neurodegenerative disease, Retinal imaging, Longitudinal studies

## Abstract

**Background:**

Artificial intelligence (AI) that utilizes deep learning (DL) has potential for systemic disease prediction using retinal imaging. The retina’s unique features enable non-invasive visualization of the central nervous system and microvascular circulation, aiding early detection and personalized treatment plans for personalized care. This review explores the value of retinal assessment, AI-based retinal biomarkers, and the importance of longitudinal prediction models in personalized care.

**Main text:**

This narrative review extensively surveys the literature for relevant studies in PubMed and Google Scholar, investigating the application of AI-based retina biomarkers in predicting systemic diseases using retinal fundus photography. The study settings, sample sizes, utilized AI models and corresponding results were extracted and analysed.

This review highlights the substantial potential of AI-based retinal biomarkers in predicting neurodegenerative, cardiovascular, and chronic kidney diseases. Notably, DL algorithms have demonstrated effectiveness in identifying retinal image features associated with cognitive decline, dementia, Parkinson’s disease, and cardiovascular risk factors. Furthermore, longitudinal prediction models leveraging retinal images have shown potential in continuous disease risk assessment and early detection. AI-based retinal biomarkers are non-invasive, accurate, and efficient for disease forecasting and personalized care.

**Conclusion:**

AI-based retinal imaging hold promise in transforming primary care and systemic disease management. Together, the retina’s unique features and the power of AI enable early detection, risk stratification, and help revolutionizing disease management plans. However, to fully realize the potential of AI in this domain, further research and validation in real-world settings are essential.

## Background

Artificial intelligence (AI) is a branch of computer science that was developed in the 1950s with the goal of creating intelligent machines [1]. Machine learning (ML), a subset of AI, involves algorithms that learn from examples rather than being manually programmed [1]. On the other hand, deep learning (DL) is built upon the artificial neural networks (ANN), which mimic the functional structure of a human central nervous system (CNS) [1]. In DL, a single deep neural network can gather data as well as learn to separate out features that are appropriate for specific classification task and then categorize them [1]. In essence, the key distinction between ML and DL lies in how they learn and process information. ML relies on algorithms to perform task without explicit programming, while DL employs a complex algorithmic structure inspired by the human brain [1].

This understanding of AI, ML and DL forms the foundation for their application in ophthalmology, particularly in the realm of retinal imaging for systemic disease prediction and diagnosis.

Artificial intelligence (AI) and DL techniques with ophthalmology have gained momentum, capitalizing on the retina’s unique role as a direct window into the CNS and microvascular circulation [2]. Retinal changes have been linked to systemic conditions like cardiovascular disease (CVD) and neurological disorders, evidenced by vascular tortuosity and retinal nerve fiber layer thinning [3, 4], prompting exploration of AI-driven retinal imaging for systemic disease prediction and diagnosis. Beyond its visual significance, the retina holds profound insights into overall health, with robust connections established between retinal findings and conditions such as hypertension, diabetes mellitus (DM), CVD, and neurodegenerative disorders including Alzheimer’s disease (AD) [5–7]. This underlines the retina’s potential as an invaluable diagnostic tool for early detection and intervention, emphasizing its pivotal role in reshaping disease assessment and risk evaluation.

In essence, a retinal biomarker is an objective measure used in predicting, evaluating, diagnosing, and planning treatment for various medical conditions. It is essential to note that this concept of biomarkers predates the work of Cheung et al. [8], who made a notable contribution in elaborating on the concept. DL is transforming the field of retinal biomarkers by utilizing extensive datasets and powerful computational algorithms to derive valuable insights from retinal imaging [8]. DL proficiency in learning intricate image features leads to the developing of a “retinal fingerprint” for diseases [8]. This profound analysis of retinal images empowers DL models to construct robust predictive frameworks for systemic diseases, revolutionizing disease detection and offering a powerful tool for precise diagnostics [8].

Central to AI’s potential is its longitudinal predictive prowess, which holds distinct advantages in the shift towards value-based healthcare. Utilizing baseline retinal photos, longitudinal prediction models forecast the likelihood of future systemic diseases such as CVD and chronic kidney disease (CKD) [9]. By continuously assessing disease risk, these models enable timely interventions, personalized treatments, and optimized patient outcomes [10]. AI’s unparalleled ability for longitudinal prediction lies in its innate capacity to uncover hidden trends and subtle shifts that evade human perception [10].

Unlike cross-sectional prediction reliant on single data point, longitudinal prediction quantifies and anticipates disease progression, thereby transforming disease management and heralding a new era of precision medicine [10]. AI has demonstrated significant promise in quantifiable risk assessment in specific contexts, where DL models have been rigorously compared to human assessment, indicating its potential to enhance disease prediction and management strategies [11].

Here, we aim to explore the traditional value of the retina for systemic disease assessment, examine the potential of AI-based retinal biomarkers in predicting various systemic diseases, and emphasize the importance of longitudinal prediction models for early detection and personalized care. We will review relevant studies that have utilized DL algorithms on longitudinal data to forecast the incidence of systemic diseases, including hypertension, DM, CVD, AD, Parkinson’s disease (PD), and CKD. By understanding the current landscape and challenges in this emerging field, we can pave the way for future advancements and applications of AI in ophthalmology for improved patient care.

## Main text

Electronic bibliographic searches in PubMed and Google Scholar up to 20 June 2023 were carried out for this narrative review. MeSH terms and all-field search terms were searched for the following criteria: “artificial intelligence”, “deep learning”, “systemic disease”, “cardiovascular disease”, “neurodegenerative disease”, “retinal imaging”, “eye”, and “longitudinal”, “fundus photographs”. The search was supplemented further by using references listed in the publications that were identified. We excluded abstracts, correspondence, opinions, editorials, letters, cross-sectional studies, and studies involving optical coherence tomography (OCT) scans from our selection. Only papers in the English language were used in this review.

Data extracted include study setting details (study name, first author, year of publication, study design, study type, adjusted variables in the model), study population (sample size, internal dataset, and external dataset), application (name of systemic disease, disease category, outcome formality, definition of the retinal biomarker), AI model used (name of the neural network, training platform), study results and their conclusions (Tables 1, 2 and 3).

**Table 1:** Summary of studies in the current literature (neurodegenerative diseases)

Author	Study year	Key findings	Retinal biomarker	Dataset	Total No. of participants	Adjusted variables	Study design
Cheung et al. [12]	Between August 2010 and December 2018	Narrower retinal arteriolar calibre and wider retinal venular calibre were associated with an increased risk of cognitive decline and incident dementia.	SIVA-DLS	Participants from National University Hospital and St Luke’s Hospital in Singapore	491	Age Gender Ethnicity Education Cerebrovascular disease status Hypertension Hyperlipidaemia Diabetes Smoking	Prospective cohort study
Hu et al. [13]	Between 2006 and 2010	Each one-year increase in retinal age gap was independently associated with a 10% increase in the risk of incident PD.	Retinal age gap	UK Biobank	35,834	Age Gender Ethnicity Townsend deprivation Smoking status Drinking status Obesity Physical activity History of stroke History of diabetes mellitus Hypertension Use of psychotropic Medication	Prospective cohort study
Wagner et al. [14]	Between 1st January 2008 and 1st April 2018	Patients with AMD and DR were at a higher risk of developing AD compared to those without these eye diseases. These findings suggest that ophthalmic imaging (e.g., OCT and fundus photography) could be used as non-invasive tools for early detection and monitoring of AD.	Retinal photographs	Moorfields Eye Hospital in London, UK	353,157	Age Gender Ethnicity	Retrospective cohort study
Cheung et al. [15]	-	The DL model achieved high accuracy in detecting AD in testing datasets, even when the datasets were from different populations and had different imaging protocols. The system could be used as a non-invasive, low-cost, and scalable tool for AD screening and monitoring.	Retinal photographs	MACC Novel Retinal Imaging Biomarkers for Cognitive Decline, Hong Kong Queen’s University of Belfast, UK SEED HKCES University of Hong Kong Volunteer Cohort	3,888 (648 with AD, 3240 without AD)	Age Gender Presence of hypertension Presence of diabetes	Retrospective multicenter case control study

*AMD = *age-related macular degeneration; *AD = *Alzheimer’s disease; *DL = *deep learning; *DR = *diabetic retinopathy; *HKCES = *Hong Kong Children Eye Study; *MACC = *Memory, Ageing and Cognition Center; *OCT = *optical coherence tomography; *PD = *Parkinson’s disease; *SEED = *Singapore Epidemiology of Eye Diseases; *SIVA-DLS = *Singapore I Vessel Analyzer Deep-Learning System

**Table 2:** Summary of studies in the current literature (cardiovascular disease)

Author	Study year	Key findings	Retinal biomarker	Dataset	Total No. of events/ participants	Adjusted variable	Study design
Rim et al. [16]	-	Reti-CAC is a reliable and effective alternative to CT scan-measured coronary artery calcification (CAC) for predicting cardiovascular events. Adding Reti-CAC to the PCE enhances cardiovascular risk stratification, evidenced by a continuous net reclassification index of 0.261.	Reti-CAC score	Severance Main Hospital, Seoul, South Korea CMERC­HI (South Korea) SEED (Singapore) UK Biobank (UK)	**527 (CMERC-HI)** 33 (6.3%) of 527 had CVD events during the 5-year follow-up **8,551 (SEED)** 310 (3.6%) of 8551 had CVD events **47,679 (UK Biobank)** 337 (0.7%) of 47679 had CVD events	Age Gender Hypertension Dyslipidaemia Diabetes Smoking status BMI Fasting glucose level	Retrospective cohort study
Tseng et al. [17]	May 2021	Reti-CVD, derived from retinal photographs, indicates a higher CVD risk, and serves as an effective tool for identifying individuals with a ≥ 10% 10-year CVD risk, including those in the borderline-QRISK3 category. It accurately identifies high-risk individuals, with a 10-year risk of 13.1% (CI: 11.7%–14.6%), enhancing early intervention and refining risk assessments, especially for those with a 10-year risk of 7.5%–10%.	Reti-CVD score	UK Biobank	48,260 (6.3% of 48,260 had CVD events during the 11-year follow-up)	Age Gender Smoking status Body mass index Hypertension Total cholesterol level Diabetes mellitus status	Retrospective cohort study
Chang et al. [18]	Between January 2005 and December 2017	DL-FAS, using retinal fundus images, predicts CVD death risk, adding value beyond the Framingham risk score (FRS). It indicates atherosclerosis severity with a DL-FAS > 0.66 marking a significantly higher CVD death risk (HR 8.33) compared to DL-FAS < 0.33. Enhancing FRS models, DL-FAS raises concordance by 0.0266 and stands as an independent CVD death predictor.	DL-FAS	HPC-SNUH	32,227	Age Gender BMI FRS risk level Smoking status Alcohol consumption Exercise Diabetes status Hypertension Dyslipidaemia	Retrospective cohort study
Cheung et al. [19]	January 2005 and December 2016	Retinal-vessel calibre measurements from DL models correlate with CVD risk, matching or surpassing expert graders in predictive accuracy. These models achieve high precision in retinal-vessel measurement, with intraclass correlation coefficients of 0.82 to 0.95 compared to expert assessments.	SIVA-DLS	SEED Dataset	59,191	Age Gender Ethnicity MABP BMI Total cholesterol level HbA1c Smoking status	Retrospective cohort study
Poplin et al. [20]	UK Biobank –2006 to 2010 EyePACS – 2007 to 2015	DL models using retinal fundus images accurately predict the onset of MACE within 5 years and quantify previously elusive cardiovascular risk factors (age, gender, smoking status, blood pressure). Predictive accuracies include age with mean absolute error 3.26 years, gender (AUC: 0.97), smoking status (AUC: 0.71), systolic blood pressure with mean absolute error 11.23 mmHg, and MACE (AUC: 0.70).	Retinal fundus images	UK Biobank EyePACs Dataset	12,026 patients, 91 with prior cardiac events were excluded. In the remaining 11,835 patients, 105 experienced MACE within 5 years post-retinal imaging.	Age Gender Smoking status HbA1c Blood pressure BMI Major adverse Cardiovascular events	Observational cohort study

*AUC = *area under the curve; *BMI = *body mass index; *CI = *confidence interval; *CMERC­HI = *Cardiovascular and Metabolic Disease Etiology Research Center-High Risk; *CT = *computational tomography; *CVD = *cardiovascular disease; *DL-FAS = *deep learning-funduscopic atherosclerosis score; *HbA1c = *hemoglobin A1c; *HR = *hazard ratio; *HPC-SNUH = *Health Promotion Center of Seoul National University Hospital; *MACE = *major adverse cardiovascular events; *MABP = *mean arterial blood pressure; *PCE = *pool cohort equation; *SEED = *Singapore Epidemiology of Eye Diseases

**Table 3:** Summary of studies in the current literature (chronic kidney disease)

Author	Study type	Study year	Key findings	Retinal biomarker	Dataset	Total of events/ participants	Adjusted variable	Study design
Zhang et al. [21]	CKD	September 2019 to November 2019	DL models analysing retinal photos and clinical data effectively detect CKD and T2DM, predict key health markers like eGFR and blood-glucose levels, and risk-stratify patients. With AUCs of 0.85–0.93, these models accurately identify CKD and T2DM, showcasing potential in disease progression risk stratification.	Retinal Fundus Images	CC-FII	Cross-sectional Cohort CC-FII: 3,156 Longitudinal dataset CC-FII-L: 10,269 External longitudinal test set: 3,376 External test set 1: 8,059 External test set 2: 3,081	Age Gender Blood pressure Height Weight BMI Hypertension T2DM	Prospective, Retrospective, Longitudinal
Joo et al. [22]	CKD	UK Biobank (each participant was followed up to 11.6 years from their initial visit to the last date of visit Feb 28, 2021) Korean Diabetic Cohort (each participant was followed up to 14 years from the data of initial visit to the last date of visit Feb 28, 2022)	Higher Reti-CKD scores correlate with increased CKD risk and outperform eGFR methods in stratifying future risk among those with normal kidney function. In the highest *vs.* lowest quartile, hazard ratios were 3.68 (UK Biobank) and 9.36 (Korean Diabetic Cohort), indicating superior Reti-CKD predictive accuracy.	Reti-CVD	UK Biobank Korean Diabetic Cohort	30,477 UK Biobank 5,014 Korean Diabetic Cohort	Age Gender Diabetic status Hypertension status eGFR	Observational cohort study
Zhang et al. [23]	CKD	2006 to 2010	Every one-year increase in the retinal age gap (model-based retinal age *vs.* chronological age) is associated with a 10% higher kidney failure risk, highlighting its potential as a non-invasive biomarker. This is based on a DL model analysing retinal images against chronological age.	Retinal age gap	UK Biobank	500,000	Age Gender Ethnic background Townsend deprivation indices (TDI) Smoking status Alcohol consumption Physical activity level General health status Diabetic status Blood pressure BMI Cholesterol level eGFR	Prospective cohort study

*AUC = *area under the curve; *BMI = *body mass index; *CC-FII = *China Consortium of Fundus Image Investigation; *CKD = *chronic kidney disease; *CVD = *cardiovascular disease; *DL = *deep learning; *eGFR = *estimated glomerular filtration rate; *T2DM* = type 2 diabetes mellitus

### Neurodegenerative diseases

The retina shares developmental origins, anatomical features, and physiological properties with vital organs, such as the brain. It can be considered an extension of the CNS [24, 25]. These properties include microvascular architecture, regulation of blood flow, the function of vascular barriers, and the crucial role of neurovascular coupling responses in maintaining homeostasis [24, 25]. The intimate connection between the CNS and the microcirculation in the brain also has a significant and direct influence on the microcirculation within the retina [24, 25]. This holds significant implications for the detection and understanding of various neurodegenerative conditions, including PD and AD, which primarily affect the brain and spinal cord [26]. Research studies have shown that certain ocular indicators can be correlated with early-stage cognitive impairment, shedding light on the potential role of ocular assessments in the early detection of cognitive decline [27]. Thinning of the retinal nerve fiber layer has been observed in individuals with cognitive impairment, suggesting a potential link between retinal changes and early-stage cognitive decline [15]. An investigation into the visual abnormalities in PD have provided valuable insights [28]. Extensive research indicates that retinal dopamine deficiency significantly contributes to the visual impairments experienced by PD patients, including deficits in acuity, contrast sensitivity, and color perception. This is supported by evidence such as reduced dopamine innervation around the fovea [27], decreased retinal dopamine concentration, thinner inner retinal layers, reduced retinal electrical activity [28–31]. Additionally, the presence of misfolded α-synuclein, a hallmark of PD, in the inner retinal layer further supports the notion of retinal involvement in the disease [28, 32]. Other studies have also shown that OCT-measured retinal thickness is associated with frontal temporal lobe dementia and the severity of the disease is correlated with retinal thinning [33].

### Role of retinal biomarkers in neurodegenerative disease without AI

Invasive techniques were used in the early diagnosis of AD, including positron emission tomography (PET) scans and cerebrospinal fluid (CSF) analysis, which pose a risk to patients [34]. These methods are expensive and have poor sensitivity, often necessitating postmortem histological examination for a conclusive diagnosis [35]. Recent studies, however, suggest that non-invasive methods, notably retinal imaging, can be extremely useful in identifying AD.

Retinal imaging techniques such as OCT, optical coherence tomography angiography (OCTA), and dynamic vessel analysis have emerged as promising non-invasive methods to investigate functional and structural retinal biomarkers associated with AD and vascular cognitive impairment and dementia [36]. Retinal imaging allows for a non-invasive examination of the anatomical and functional changes impacting the brain, making AD detection more practical and affordable.

In the last two decades, significant progress has been made in the field of retinal imaging. The development of semiautomated software has enabled more precise quantitative measurement of retinal vessel calibres from retina fundus photographs. This non-invasive approach has proven to be extremely valuable in identifying AD and exploring its association with cognitive function [7, 35, 37–39]. However, most of these studies often adopt a cross-sectional approach design [7, 35, 37–39], with only a few longitudinal studies [40] investigating the detection of subtle changes in the link between retinal vessel calibres and the risk of cognitive decline and dementia [40]. Consequently, the absence of longitudinal data hinders the ability to draw definitive conclusions.

Furthermore, the use of semiautomated retinal vessel measurement software has its own limitations. This software heavily relies on human input and is a time-consuming and error prone procedure [41, 42]. As a result, the variability in measuring retinal vessel calibre could have contributed to inconsistent findings [41, 42].

### AI-driven retinal biomarkers for neurodegenerative diseases

AI-based retinal biomarkers have emerged as a promising approach for the early detection and monitoring of neurodegenerative diseases. Several studies have demonstrated the effectiveness of AI-based approaches in predicting and diagnosing neurodegenerative diseases using retinal imaging data (Table 1). A prospective study conducted by Cheung et al. [12], which utilized a DL algorithm on retinal photographs, investigated the relationship between DL retinal vessel calibre measurement and the risk of cognitive decline and dementia [12]. Their study adds to the growing body of evidence that narrow retinal arteriolar calibre at baseline is associated with an increased risk of cognitive decline, and it is also found to be predictive of future dementia development [12]. This longitudinal approach highlights the potential of retinal imaging and vessel assessment as non-invasive tools for early screening and stratification of individuals susceptible to cognitive decline and dementia.

In another study, a DL algorithm was employed to examine the association between the retinal age gap (retinal age–chronological age) and the risk of developing PD measured through retinal images. The finding revealed that an increase of one year in the retinal age gap was independently associated with a 10% higher risk of PD [13, 43]. The study’s results highlighted the potential of the retinal age gap, measured using a DL algorithm, as a promising biomarker for identifying individuals at a higher risk of developing PD. The use of the retinal age gap, a non-invasive and cost-effective measure obtained through retinal imaging, offers an opportunity for large-scale screening. However, this study’s limitations include selection bias due to a healthier and younger participant sample from the UK Biobank, limited incident PD cases for subgroup analysis, the absence of longitudinal retinal age gap data, and the possibility of unaccounted residual confounders [13].

Additionally, the AlzEye study [14] aims to integrate longitudinal retinal imaging data from Moorfields Eye Hospital NHS Foundation Trust with systemic disease data from hospital admissions. By linking these datasets together, the primary focus of the analysis will be on CVDs and dementia, with the objective of uncovering hidden retinal signatures that can facilitate earlier detection and risk management [14]. AlzEye study’s limitations include potential biases from reliance on hospital admission data, which may not fully capture the general population and could lead to under-recording of crucial variables as well as the inherent selection bias associated with the AlzEye cohort, which consists of individuals with definite or suspected ophthalmic disease, potentially limiting the external validity of the findings [14].

The work conducted by Cheung et al. [15] presents a significant breakthrough in the field of AD detection. Traditional methods for diagnosing AD are known for their complexity and invasiveness, often involving PET scans, CSF collection, and plasma assays to measure biomarkers such as amyloid β and phosphorylated tau [15, 44]. In contrast, this study introduced a novel approach that harnesses the capabilities of DL, specifically tailored to analyse retinal photographs [15]. The study’s methodology involved the compilation of data from 11 separate studies, amalgamating retinal images from both AD patients and healthy subjects. During the model’s internal validation, impressive results were achieved, with accuracy of 83.6%, sensitivity of 93.2%, specificity of 82.0%, and an area under the receiver operating characteristic curve (AUROC) of 0.93 [15]. Subsequent testing across diverse datasets demonstrated accuracy levels ranging from 79.6% to 92.1%, accompanied by AUROCs spanning from 0.73 to 0.91 [15]. Additionally, the DL algorithm exhibited the capability to distinguish between participants with positive and negative amyloid β status [15]. This research signifies a pivotal advancement in AD screening, providing an innovative and non-invasive means of early detection using retinal images [15]. However, the study has limitations, including a small training dataset, potential labelling inaccuracies in the clinician-derived diagnosis, unaccounted biases, and variable model performance across testing cohorts [15]. Nonetheless, it represents a groundbreaking approach in AD detection using retinal images, offering a promising non-invasive screening method [15].

### Cardiovascular diseases (CVD)

The retina shares embryological, anatomical, and physiological characteristics with vital organs such as the brain and kidneys, making it a valuable source of information about the systemic microvasculature [45]. Through the analysis of retinal vessels, which indirectly reflect the state of the systemic microvasculature, valuable information can be obtained concerning microvascular alterations that commonly occur prior to the development of macrovascular disorders such as stroke and ischemic heart disease [46].

### Role of retinal biomarkers in CVD without AI

Retinal studies have revealed associations between retinal changes and various systemic CVDs. For instance, diabetic retinopathy (DR) and hypertensive retinopathy, both well-established retinal diseases, have been associated with premature morbidity and mortality of CVDs [47–49]. For instance, DR, which primarily affects individuals with poorly controlled diabetes and/or prolonged diabetes duration, highlights the intricate relationship between retinal alterations and systemic health. By identifying and characterizing these retinal biomarkers, clinicians gain insights into an individual’s health status and disease risk, enabling timely interventions [50, 51].

One limitation of traditional methods is the subjectivity and potential variability introduced by human visual perception. Different individuals may have varying levels of expertise and subjective interpretations when analysing retinal images. This can lead to inconsistencies and potential errors in identifying and quantifying specific retinal changes associated with CVDs [19].

In summary, traditional retinal studies helped lay the foundation for understanding the association between retinal changes and systemic diseases, including CVD. These studies have demonstrated the potential of retinal imaging as a non-invasive tool for early detection, risk assessment and monitoring of various systemic conditions.

### AI-driven retinal biomarkers for CVD

Research incorporating DL techniques demonstrate promising outcomes in predicting and diagnosing CVD through retinal imaging. CVD events were characterized as instances of hospitalization or mortality resulting from specific conditions such as myocardial infarction, stroke, unstable angina, transient ischemic attack, peripheral vascular disease, acute coronary heart disease, as well as procedures including coronary, carotid, or peripheral artery revascularizations, and major associated amputations [52]. For instance, DL showcases a robust correlation between fundus image features and CVD risk [52], introducing a new dimension to the existing comprehension of retinal biomarkers. Notably, integrating a DL score into predictive models along with conventional clinical risk factors subtly yet significantly enhances the prediction of CVD risk for individuals with diabetes [52].

One study from Table 2, by Rim et al., developed a DL-based algorithm to predict cardiovascular risk using retinal photographs [16]. Their algorithm, RetiCAC, outperformed single clinical parameters, such as age, glucose, or smoking status in predicting the presence of coronary artery calcium (CAC) [16] with an AUC of 0.742 [16, 17]. Additionally, the study evaluated the synergy between RetiCAC and the pool cohort equation (PCE), a well-established risk stratification framework endorsed by the American College of Cardiology/American Heart Association (ACC/AHA) guidelines [53, 54]. When integrating RetiCAC with PCE, the researchers observed an improved risk stratification for individuals classified within the intermediate and borderline risk groups. The study suggests that retinal photograph-based DL can serve as an alternative measure of CAC, particularly in low-resource settings [16, 17]. Additionally, it is notable that the study acknowledges its own limitations. The study encompasses a diverse range of ethnicities, including those in Singapore (comprising predominantly Chinese, Malay, and Indian populations), South Korea, and the United Kingdom, but broader ethnic representation could enhance its validity [16]. A following study led by Tseng RMWW et al. validates Reti-CVD (formerly RetiCAC) as a promising biomarker for identifying individuals with a 10% or higher 10-year CVD risk and enhancing risk assessment for those in the borderline group (risk of 7.5%–10%). This is particularly relevant when considering traditional risk calculators like QRISK3, a clinical algorithm used to estimate 10-year CVD risk [17]. This study emphasizes the potential of Reti-CVD to advance cardiovascular risk stratification [17].

In another study, Diaz-Pinto et al. [55] developed a system that utilizes retinal photographs and patient demographic data to estimate cardiac indices [55]. The study presents a system that has a potential in predicting future myocardial infarction (MI) events during routine ophthalmic visits, by estimating the left ventricular mass (LVM) and left ventricular end-diastolic volume (LVEDV) [55]. The ability to predict forthcoming MI events from retinal images in the UK Biobank population demonstrated a sensitivity of 0.74, specificity of 0.72, and precision/positive predictive value (PPV) of 0.68 when considering only age and gender as supplementary demographic factors [55]. In the Age-Related Eye Disease Study (AREDS) population, the approach’s sensitivity, specificity, and precision/PPV for predicting future MI events from retinal images were 0.70, 0.67, and 0.67, respectively, following the exclusion of all age-related macular degeneration (AMD) cases [55]. By incorporating cardiac indices and demographic data, the system demonstrated improved accuracy in predicting MI compared to using demographic data alone [55].

Cheung et al. [19] conducted a comprehensive study on the use of DL models to automatically measure retinal vessel calibre in retinal photographs, aiming to evaluate the correlation with CVD risk [19]. The research involved a diverse dataset with a substantial number of images collected from various ethnicities and countries. The DL models exhibited strong agreement with expert human graders in accurately measuring retinal vessel calibre [19]. Moreover, the models demonstrated comparable or superior performance to human graders in associating retinal vessel calibre with key CVD risk factors, including blood pressure, body mass index, total cholesterol, and glycated haemoglobin levels [19]. Notably, the study revealed that the initial measurements obtained through the DL system were prospectively linked to incident CVD in retrospectively analysed datasets. However, the study by Cheung et al. [19] has several limitations. They only trained and tested the DL model on gradable retinal photographs, potentially excluding ungradable images that could provide valuable data [19]. Additionally, the study relied on human measurements as ground-truth labels, introducing the possibility of intergrader variability affecting the model’s performance and accuracy [19].

In the study by Rudnicka AR et al. [20], the authors aimed to enhance the understanding of the relationship between retinal vasculometry (RV) and CVD risk by developing an algorithm that utilized DL methods to distinguish between arterioles and venules, and thus incorporating AI-enabled retinal vasculometry as an alternative biomarker [20]. This AI-based retinal vasculometry employed a fully automated system known as QUARTZ [20]. QUARTZ utilized a supervised ML model to create an image quality score, and DL algorithms were used to distinguish between arterioles and venules [20]. The study compared the performance of Framingham risk score (FRS) for incident stroke and MI with the addition of RV to FRS, as well as a simpler model based on RV, age, smoking status, and medical history. However, the addition of RV to FRS did not improve the prediction of incident stroke and MI in either cohort [20]. Interestingly, the simpler RV model performed equally or better than FRS [20]. The study concluded that RV could serve as an alternative predictive biomarker for vascular health without the need for invasive blood sampling or blood pressure measurement [20]. However, it is important to acknowledge the study’s limitations, including its reliance on cohorts who are considered healthy with low event rates and a predominantly White population, which may affect the generalizability of the findings [20]. Further validation in more diverse and high-risk cohorts is needed to confirm its applicability in broader populations [20].

Another study by Poplin et al. [56] demonstrated the potential of DL models to extract cardiovascular risk factors from retinal photographs. The results showed that the DL models were able to accurately predict various risk factors, including age, gender, smoking status, systolic blood pressure, and major adverse cardiac events with AUC 0.73 [95% confidence interval (CI):0.690.77] [56]. These predictions were based on anatomical features present in the retina, such as blood vessels and the optic disc. However, the study acknowledges its limitations, including a relatively small dataset with narrow field of view images and missing essential clinical inputs, emphasizing the need for validation on larger, more diverse datasets to enhance the accuracy and generalizability of their DL models [56].

A study conducted by Chang et al. [18] created a DL model that could predict atherosclerosis using retinal images and examined its clinical complications. The findings showed that individuals with higher DL-funduscopic atherosclerosis score (FAS) had an increased risk of CVD disease related deaths compared to those with lower DL-FAS scores [18]. The DL-FAS also improved the prediction of CVD deaths when combined with the Framingham risk score (FRS), a commonly used risk assessment tool [18]. However, the limitation of the study is that it is a single-center database which comprised solely of South Koreans, and thus limits its generalizability, a critical concern given the dependency of CVD risk on ethnicity [57].

### Chronic kidney disease (CKD)

CKD frequently presents insidiously, with patients typically remaining asymptomatic during the early stages for prolonged periods, leading to low awareness of the condition [58]. However, as the disease progresses, patients may experience symptoms such as polyuria or fatigue due to anaemia, highlighting a critical stage where the risk of complications and progression to end-stage renal disease (ESRD) significantly increases [59]. The assessment of kidney function primarily relies on glomerular filtration rate (GFR) measurements, often calculated using serum creatinine concentration through specific formulas such as the Chronic Kidney Disease Epidemiology Collaboration (CKD-EPI) equation [59]. Additionally, CKD can be detected by abnormal results in routine blood tests, such as elevated urea nitrogen, cystatin C levels, and the presence of protein or albumin in urine [59]. By harnessing DL’s analytical capabilities for retinal imaging, which shares, anatomical, embryological, and physiological characteristics with vital organs such as the kidneys [45], researchers can explore the potential link between retinal microvascular alterations and early stages of CKD. These findings hold promise for uncovering novel prognostic markers and risk stratification tools, empowering clinicians to intervene early and mitigate the burden of CKD-related complications.

### Prior research on retinal biomarkers without AI

Prior to the use of AI, studies examined the relationship between elevated blood urea nitrogen and creatinine levels and the occurrence of specific eye conditions, including posterior subcapsular cataract [60], late AMD [61], and DR [62], suggesting potential associations with kidney function changes. Conversely, alterations in retinal signs have been observed to potentially indicate changes in kidney function. The utilization of AI can help early identification with retinal imaging and can help identify and minimize vascular damage to the kidneys.

### AI-driven retinal biomarkers for CKD

The longitudinal studies that evaluate retinal biomarkers that can be utilized for CKD assessment have been summarized in Table 3. Zhang et al. [21] utilized a DL-based model to assess the risk of progressing to advanced (stage 3) and severe (stages 4 and 5) CKD over a span of six years within a longitudinal cohort. Additionally, their research, as presented in Table 3, focused on predicting the development of CKD in the same cohort, incorporating baseline retinal imaging and clinical metadata [21]. The findings revealed that the combined model, which integrated risk scores extracted from retinal images and clinical metadata, demonstrated significantly enhanced predictive performance compared to utilizing clinical metadata alone [21]. This indicates the potential of retinal images as a valuable screening tool for risk assessment and personalized treatment in the context of CKD. This was seen in the model, where the AI was used in the identification of type 2 diabetes mellitus (T2DM) using retinal images of T2DM from healthy controls with high area under the curve (AUC) values for the metadata-only model (AUC = 0.828), the fundus image-only model (AUC = 0.923), and the combined model (AUC = 0.929) on the internal test set [21]. Additionally, the DL model successfully stratified patients into low-, medium- and high-risk groups for developing CKD [21]. This demonstrates the potential for early detection and risk stratification using AI-based retinal biomarkers. However, training of the DL model was limited to a predominately Chinese population [21]. The study can benefit from additional validation with an external multi-ethnic population [21].

Additionally, in another study by Zhang et al. [23], who developed a DL model to assess retinal age, the difference between model-based retinal age and chronological age, termed the retinal age gap, was used to predict the risk of ESRD. Through Cox proportional hazards regression models, they observed that a one-year increase in the retinal age gap corresponded to a 10% rise in the risk of incident ESRD [hazard ratio (HR) = 1.10, 95% CI: 1.03–1.17] [23]. Given the suitability of retinal images for early prediction and longitudinal assessment, the study not only provided valuable data for the estimation of the progression of ESRD but also served as a predictive indicator of mortality [23]. Therefore, retina images have the capability to serve as a screening method for evaluating risk and providing individualized treatment. In tandem with previously mentioned studies that utilized the UK Biobank cohort, this study also exhibits limitations, including a restricted subgroup analysis due to a small number of patients with kidney failure and the absence of longitudinal fundus photography data, which may impact the generalizability and depth of findings [23].

Last, a study by Joo et al. [22] developed a non-invasive CKD risk stratification tool called “Reti-CKD” derived from retina-based DL and clinical factors [22]. The performance of the Reti-CKD was compared against traditional estimated glomerular filtration rate (eGFR) based methods that assess the kidneys’ ability to filter toxins or waste from our blood [22]. When compared to the current standard of care (eGFR-CKD score), the Reti-CKD score exhibited significantly greater predictive performance based on C-statistic and net reclassification index (NRI) values [22]. Overall, the study showcases the potential of an AI-based biomarker, the Reti-CKD score, in a non-invasive way for predicting the risk of CKD development by leveraging DL algorithms trained on retinal photographs and incorporating clinical factors [22]. The Reti-CKD score outperformed traditional eGFR-based methods [22]. Lastly, external validation of this study was limited due to the Korean Diabetic Cohort, warranting the need of further validation in diverse disease populations and ethnicities [22].

### Overall limitations of AI in retinal biomarkers

While AI holds promise in retinal imaging for systemic disease prediction, its practical application faces limitations. Robust models demand extensive and diverse datasets, highlighting the challenge of bias-free data collection. Moreover, the potential of longitudinal prediction models for personalized treatment plans is hindered by scarce longitudinal data for training and validation. Thus, capturing disease progression over time is essential for systemic disease prediction. Additionally, unaccounted confounding factors, such as pulse cycle-induced retinal calibre variations, medical history (such as hypertension and diabetes), medications, and distinct individual retinal pathologies, can impact outcomes [12]. Notably, some studies exhibit imbalanced distributions of clinical conditions and demographics among participants, potentially distorting relationships between retinal biomarkers (e.g., retinal vessels calibre) and neurodegenerative conditions, leading to misleading conclusions [12].

There are ethical concerns surrounding the use of AI in healthcare. An example would be the technical limitation of AI utilization, stemming from the fact that AI-based systems frequently suffer from a lack of transparency [63]. While it was once common to label DL as black boxes due to their limited explainability [63, 64], the field of explainable artificial intelligence (XAI) has made significant progress in recent years [65]. Today, there are numerous XAI methods that have been developed to address the issue of model transparency and interpretability [65]. These methods provide insights into how AI models arrive at a specific prediction, enhancing our ability to understand their output [65]. One example in the context of cardiac imaging studies is the application of post hoc interpretability methods such as “gradient-weighted class activation mapping (Grad-CAM)” which has been proven invaluable [65]. Grad-CAM generates heatmaps that visually reveal which specific areas within a medical image have influenced the AI model’s diagnostic decision. By highlighting the regions that played a pivotal role in the model’s output, these heatmaps offer clinicians and researchers a clear and interpretable representation of how AI algorithms arrive at their conclusions [65].

In the context of AI investigations within the field of ophthalmology, there are various limitations that affect the development and application of AI algorithms for retinal biomarkers. The issue relates in how potential biases, geographical skew, and stakeholder diversity significantly impact the development of guidelines and recommendations [66]. One example of this representation is the Developmental and Exploratory Clinical Investigations of Decision support systems driven by Artificial Intelligence (DECIDE-AI) survey which was heavily skewed towards European and UK scientists (83% of scientific experts) [66]. This poses a significant hurdle for generalizability of AI algorithms when used in different environments [66]. This is because the AI systems developed were highly dependent on their operational environment and their performance can be affected in different settings [67]. This bias in representation can lead to challenges in ensuring the broad applicability of AI algorithms in different ophthalmological settings [67].

Similarly, the lack of randomized controlled trials (RCTs) for comparison in the performance of AI models with the current standard of care can impede the integration of AI into clinical practice [68].

There is also difficulty in ensuring good quality and consistency of retinal images across different datasets [17]. This limitation underscores the need for robust quality control measures to enhance the reliability and reproducibility of AI predictions based on retinal imaging [17].

Lastly, in our comprehensive review, which focused on the application of AI-based retinal biomarkers in predicting systemic disease, we acknowledge a limitation pertaining to the exclusion of studies related to OCT imaging. While we recognize the significance of OCT as a valuable imaging modality for assessing retinal health and its potential contributions to understanding systemic disease prediction, we deliberately chose not to include it as a search criterion in our review for several reasons. First, our research scope was primarily oriented towards studies that utilize retinal fundus photography given its wide availability and non-invasive nature [69]. Second, colour fundus photography (CFP) offers practicality and accessibility in ophthalmology and primary care settings, making it the preferred tool for screening, especially in resource-limited environments. Third, its simplicity, cost-effectiveness, and ease of use distinguishes it from OCTA which requires specialized equipment and expertise, limiting its widespread use, particularly in family or internal medicine clinics [70]. Fourth, CFP remains a valuable tool for ophthalmic diagnosis because it provides information beyond microvascular circulation. CFP allows for the assessment of colour, reflexes, and signs such as the copper wiring sign in hypertensive retinopathy [71], which are essential for a comprehensive evaluation and diagnosis. These clinical features cannot be fully replicated by OCTA [71].

In the future, we could explore the synergies between OCT and retinal fundus photography in AI-driven systemic disease prediction.

## Disscusion

The clinical relevance of retinal imaging for systemic diseases is multifaceted. It enables early detection, allowing for timely diagnosis and intervention, all without the need for invasive procedures or extensive testing. Moreover, it significantly reduces the diagnostic burden on patients who would otherwise have to visit various specialists for a comprehensive evaluation.

A recent study conducted by the research teams from Moorfields Eye Hospital and UCL Institute of Ophthalmology illustrates the transformative potential of retinal biomarkers [72]. They identified indicators of PD an average of seven years before clinical diagnosis, representing a paradigm shift in healthcare [72]. It streamlines the diagnostic process by providing not only ophthalmologists but also physicians from various specialties such as neurologists with a non-invasive tool to aid in the early identification of systemic diseases (e.g., PD) [72]. This not only enhances patient care by enabling early intervention but also reduces the burden of multiple, often invasive, diagnostic procedures [72].

Looking ahead, the enhancement of DL model recognition capabilities in various image segmentation tasks presents a promising avenue for future research. Domain adaptation and transfer learning have shown their significance in previous studies as demonstrated by the work of Tian et al. [73]. However, the inevitability of device-based domain variations in clinical settings necessitates the development of robust domain adaptation techniques [73]. These techniques will enable DL models to perform effectively when presented with data from previously unseen databases, and thus enhance the practical utility of DL models in the field of medical image segmentation [73].

The application of transfer learning techniques can hold significant potential within the realm of retinal fundus photography. This approach involves harnessing knowledge from one task and applying it to a distinct yet related task, primarily by reusing a pre-trained model [74]. This methodology proves especially advantageous when confronted with tasks featuring limited data availability [74]. To illustrate this concept further, we can draw inspiration from a 2018 study by Kermany et al. [74]. Their application of transfer learning involved the utilization of a fraction of the data typically required by conventional DL methods for training [74]. They directed this approach towards an OCT dataset, addressing the challenge of choroidal neovascularization (CNV) and three additional classifications [74]. Remarkably, their model exhibited exceptional performance, achieving an accuracy rate of 96.6%, a sensitivity score of 97.8%, and specificity of 97.4%. These results rivalled the diagnostic proficiency of senior ophthalmologists [74].

By advancing the capabilities of DL models in the context of retinal imaging, we open the doors to transformative changes in the early detection and monitoring of systemic diseases, ultimately improving patient outcomes and reducing the burden of healthcare procedures.

While the utilization of retinal imaging is not currently a standard practice in frontline care, there are promising developments that warrant consideration. One key factor to highlight is the accessibility and affordability of retinal imaging technology. Several fully automated retinal imaging systems are already available in the market [75], and as technology advances, their cost-effectiveness is likely to improve [75]. These developments make it increasingly feasible for retinal vessel examination to become a routine adjunct for primary care doctors. For example, in primary care settings, where patients often receive initial assessments for various health concerns, retinal imaging could serve as a valuable addition to the diagnostic toolbox [75]. Imagine a scenario where a patient visits their family physician for a routine check-up. During this visit, alongside other standard evaluations, a retinal imaging scan is conducted as part of the assessment. Retinal fundus photography can provide valuable information about the patient’s overall health, including potential indicators of systemic diseases.

Lastly, generalizability is a cornerstone in the development of AI algorithms applied to medical image analysis, necessitating the inclusion of diverse subjects during development and validation through an external dataset. In our review of 14 studies, we identified two studies – Cheung et al. [15] and Rim et al. [16] – that meticulously adhered to these principles. They effectively employed well-designed DL techniques, including recent advancements like the EfficientNet architecture, and appropriate preprocessing methods, highlighting the potential for robust and reliable research outcomes in ophthalmology.

## Conclusions

In this comprehensive review, we explored the vast landscape of AI applications in the assessment of systemic diseases, with a particular emphasis on the transformative potential of retinal imaging as a predictive tool for detecting and monitoring neurodegenerative disease, CVD and CKD. The retina offers a unique opportunity for non-invasive visualization of the CNS and microvascular circulation, making it a valuable source of information for assessing overall health. Various studies have demonstrated the correlation between retinal changes and diseases such as neurodegenerative disorders, CVD, and CKD. AI-based retinal biomarkers have emerged as a powerful approach for early disease detection, risk stratification, and personalized care. Longitudinal prediction models, which utilize baseline retinal images to forecast the probability of developing specific diseases in the future, offer significant advantages in monitoring disease progression.

## Data Availability

Not applicable.

## Abbreviations

ACC/AHA: American College of Cardiology/American Heart Association
AD: Alzheimer’s disease
AMD: Age-related macular degeneration
AREDS: Age-Related Eye Disease Study
AUROC: Area under the receiver operating characteristic curve
CAC: Coronary artery calcium
CVD: Cardiovascular disease
CKD: Chronic kidney disease
CKD-EPI: Chronic Kidney Disease Epidemiology Collaboration
CMERC-HI: Cardiovascular and Metabolic Disease Etiology Research Center-High Risk
CC-FII: China Consortium of Fundus Image Investigation
CMR: Cardiovascular magnetic resonance
CNS: Central nervous system
CT: Computational tomography
DECIDE-AI: Developmental and Exploratory Clinical Investigations of Decision support systems driven by Artificial Intelligence
DM: Diabetes mellitus
DR: Diabetic retinopathy
ESRD: End-stage renal disease
EPIC: European Prospective Investigation into Cancer
FAS: Funduscopic atherosclerosis score
FRS: Framingham risk score
eGFR: Estimated glomerular filtration rate
GFR: Glomerular filtration rate
HR: Hazard ratio
HKCES: Hong Kong Children Eye Study
HPC-SNUH: Health Promotion Center of Seoul National University Hospital
LVEDV: Left ventricular end-diastolic volume
LVM: Left ventricular mass
MI: Myocardial infarction
MACE: Major adverse cardiovascular events
MACC: Memory, Ageing and Cognition Center
OCT: Optical coherence tomography
OCTA: Optical coherence tomography angiography
PD: Parkinson’s disease
PCE: Pool cohort equation
RV: Retinal vasculometry
RCTs: Randomized controlled trials
SEED: Singapore Epidemiology of Eye Diseases
SIVA-DLS: Singapore I Vessel Analyzer Deep-Learning System
T2DM: Type 2 diabetes mellitus
