# Correction: Prognostic potentials of AI in ophthalmology: systemic disease forecasting via retinal imaging

**DOI:** 10.1186/s40662-024-00399-w

**Published:** 2024-08-16

**Authors:** Yong Yu Tan, Hyun Goo Kang, Chan Joo Lee, Sung Soo Kim, Sungha Park, Sahil Thakur, Zhi Da Soh, Yunnie Cho, Qingsheng Peng, Kwanghyun Lee, Yih-Chung Tham, Tyler Hyungtaek Rim, Ching-Yu Cheng

**Affiliations:** 1 Cork University Hospital, Cork, Ireland; 2 Division of Retina, Severance Eye Hospital, Yonsei University College of Medicine, Seoul, South Korea; 3 Division of Cardiology, Severance Cardiovascular Hospital, Yonsei University College of Medicine, Seoul, South Korea; 4 Singapore Eye Research Institute, Singapore National Eye Centre, Singapore, Singapore; 5 Department of Ophthalmology, Yong Loo Lin School of Medicine, National University of Singapore, Singapore, Singapore; 6 Mediwhale Inc., Seoul, Republic of Korea; 7 Department of Education and Human Resource Development, Seoul National University Hospital, Seoul, South Korea; 8 Department of Ophthalmology, National Health Insurance Service Ilsan Hospital, Goyang, Republic of Korea; 9 Centre for Innovation and Precision Eye Health, Yong Loo Lin School of Medicine, National University of Singapore, Singapore, Singapore; 10 Ophthalmology and Visual Sciences Academic Clinical Program, Duke-NUS Medical School, Singapore, Singapore

**Correction: Eye and Vision (2024) 11:17** 10.1186/s40662-024-00384-3

After publication of this article [1], it was brought to our attention that a co-author Kwanghyun Lee was missed to add in the author list and the affiliation 10 below was missed in the original paper, the correct author list and authors affiliations are shown below:

Yong Yu Tan^1^, Hyun Goo Kang^2^, Chan Joo Lee^3^, Sung Soo Kim^2^, Sungha Park^3^, Sahil Thakur^4^, Zhi Da Soh^4,5^, Yunnie Cho^6,7^, Qingsheng Peng^4^, Kwanghyun Lee^8^, Yih-Chung Tham^4,^^5,^^9,10^, Tyler Hyungtaek Rim^4,6^, Ching-Yu Cheng^4,^^5,^^9,10^

^1^Cork University Hospital, Ireland

^2^Division of Retina, Severance Eye Hospital, Yonsei University College of Medicine, Seoul, South Korea

^3^Division of Cardiology, Severance Cardiovascular Hospital, Yonsei University College of Medicine, Seoul, South Korea

^4^Singapore Eye Research Institute, Singapore National Eye Centre, Singapore

^5^Department of Ophthalmology, Yong Loo Lin School of Medicine, National University of Singapore, Singapore

^6^Mediwhale Inc., Seoul, Republic of Korea

^7^Department of Education and Human Resource Development, Seoul National University Hospital, South Korea

^8^Department of Ophthalmology, National Health Insurance Service Ilsan Hospital, Goyang, Republic of Korea

^9^Centre for Innovation and Precision Eye Health, Yong Loo Lin School of Medicine, National University of Singapore, Singapore

^10^Ophthalmology and Visual Sciences Academic Clinical Program, Duke-NUS Medical School, Singapore

Besides, the Acknowledgment of grant funding should be:

We received support from the Korea Health Technology R&D Project through the Korea Health Industry Development Institute (KHIDI), funded by the Ministry of Health & Welfare, Republic of Korea, under grant number HI22C1580.

The original publication has been updated.
